# Midkine: A Promising Molecule for Drug Development to Treat Diseases of the Central Nervous System

**DOI:** 10.2174/138161211795164167

**Published:** 2011-02

**Authors:** Takashi Muramatsu

**Affiliations:** Department of Health Science, Faculty of Psychological and Physical Science, Aichi Gakuin University, 12 Araike, Iwasaki-cho, Nisshin, Aichi 470-0195, Japan

**Keywords:** Alzheimer’s disease, Cerebral infarction, Drug discovery, Glioblastoma, Ischemia, Midkine, Multiple sclerosis, Neurodegenerative diseases.

## Abstract

Midkine (MK) is a heparin-binding cytokine, and promotes growth, survival, migration and other activities of target cells. After describing the general properties of MK, this review focuses on MK and MK inhibitors as therapeutics for diseases in the central nervous system. MK is strongly expressed during embryogenesis especially at the midgestation period, but is expressed only at restricted sites in adults. MK expression is induced upon tissue injury such as ischemic brain damage. Since exogenously administered MK or the gene transfer of MK suppresses neuronal cell death in experimental systems, MK has the potential to treat cerebral infarction. MK might become important also in the treatment of neurodegenerative diseases such as Alzheimer’s disease. MK is involved in inflammatory diseases by enhancing migration of leukocytes, inducing chemokine production and suppressing regulatory T cells. Since an aptamer to MK suppresses experimental autoimmune encephalitis, MK inhibitors are promising for the treatment of multiple sclerosis. MK is overexpressed in most malignant tumors including glioblastoma, and is involved in tumor invasion. MK inhibitors may be of value in the treatment of glioblastoma. Furthermore, an oncolytic adenovirus, whose replication is under the control of the MK promoter, inhibits the growth of glioblastoma xenografts. MK inhibitors under development include antibodies, aptamers, glycosaminoglycans, peptides and low molecular weight compounds. siRNA and antisense oligoDNA have proved effective against malignant tumors and inflammatory diseases in experimental systems. Practical information concerning the development of MK and MK inhibitors as therapeutics is described in the final part of the review.

## 1 INTRODUCTION

Midkine (MK) is a heparin-binding growth factor or cytokine that promotes growth, survival, differentiation, migration and other activities of target cells [1-3]. MK plays important roles in reproduction, development and repair, and is involved in the onset and or progression of inflammatory diseases and malignancy. Therefore, both MK and MK inhibitors are expected to aid in the treatment of various diseases. 

MK is rich in both basic amino acids and cysteine, and has a molecular weight of around 13 kDa [4]. Pleiotrophin (PTN), also called heparin-binding growth-associate molecule (HB-GAM), has about 50 % sequence identity to MK [5-8]. However, the two are not closely related to other proteins. 

MK is strongly expressed during embryogenesis, especially at the midgestation stage, but is expressed only in highly restricted sites in adults [1-3, 9]. MK becomes expressed upon tissue injury and malignant transformation [1-3, 10, 11]. This restricted mode of expression makes MK a suitable target for drug development.

Here I review MK and MK inhibitors as potential therapeutics to treat diseases of the central nervous system, such as ischemic brain injury upon cerebral infarction, glioblastoma, multiple sclerosis and neurodegenerative diseases. MK and MK inhibitors will have broad areas of application and are expected to be used also to treat other diseases such as heart failure and other malignancies. 

I first describe the general properties of MK relevant to the main subject, and then focus on MK in diseases of the central nervous system and drug development. MK has been dealt with in earlier reviews [1-3, 12].

## 2 GENERAL PROPERTIES OF MK

### 2.1 Historical Background

MK was found as the product of a gene whose expression increased at early stage of the retinoic acid-induced differentiation of murine teratocarcinoma stem cells called embryonal carcinoma cells [13]. PTN was found as a factor to promote neurite outgrowth of embryonic neurons [14] or as a factor to promote growth of fibroblasts [15]. The deduced protein sequence of MK was published in 1990 [4]; that of PTN subsequently [5, 6]. A heparin-binding protein found in chicken embryos [16], called retinoic acid-induced heparin-binding [17], is the chicken form of MK. Human MK was cloned later [18, 19] and was also called heparin-binding neurotrophic factor [19] or neurite growth-promoting factor 2.

### 2.2 Protein

MK and PTN are present in all vertebrates examined so far including *Xenopus lavis* [20] and zebrafish [21]. There are two MK genes in zebrafish due to gene duplication (*Mdka* and *Mdkb*) [21]. *Drosophila melanogester* lacks them, but has miple-1 and -2, with repeated motifs common to MK and PTN [22].

**Fig. (1):**
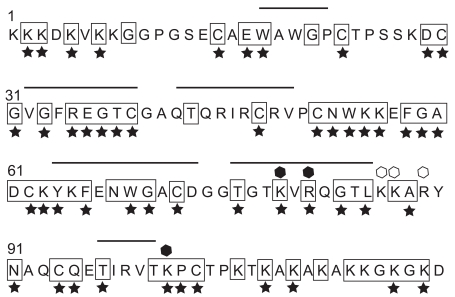
Protein sequence of human MK. Amino acids conserved between MK of different species (human MK [18], *Xenopus* MK [20] and zebrafish Mdka [21, 194]) are shown by open boxes, while those conserved between MK and PTN (human MK and PTN [6], *Xenopus* MK and zebrafish Mdka) are shown by ★. Heparin binding sites Cluster 1 and 2 are shown by closed hexagons and open hexagons, respectively. Bars show β-sheets.

Human MK and mouse MK have 87 % sequence identity [18], while human MK and *Xenopus *MK have about 60 % [20]. Positions of all 10 cysteine residues are conserved in MK of different species and also in PTN (Fig. **1**). Furthermore, tryptophane residues are present downstream of the first cysteine residues forming two of the domains of MK and PTN. This feature is the same as that of the thrombospondin type I repeat; MK as well as PTN can be considered to belong to the thrombospondin superfamily [23].

**Fig. (2):**
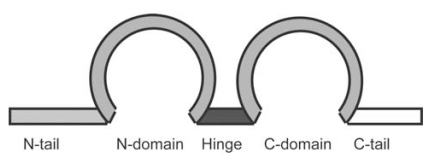
Domain structure of human MK. Homology to human PTN is more than 60 % (black color), between 40 – 60 % (grey color), or less than 40 % (white color).

MK and PTN are largely composed of two domains, the more N-terminally located N-domain and the more C-terminally located C-domain [24] (Fig. **2**). The N-domain has three disulfide linkages, while the C-domain has two. Both domains contain three antiparallel *β*-sheets [25] (Fig. **1**), but the C-domain also contains a flexible loop [25]. Besides the two domains there are an N-terminal tail (N-tail), a C-terminal tail (C-tail) and a hinge connecting the two domains (Fig. **2**). The conserved structure between MK and PTN is not confined to the two domains, but extends to the two tails and the hinge (Figs. **1,** **2**). Interestingly the hinge is the most conserved structure between MK and PTN. There is a large stretch of sequence around the hinge highly conserved between human and *Xenopus* MK and zebrafish Mdka (Fig. **1**). The tails of MK do not form stable structures, and the two domains appear to move freely to each other [25].

Deletion of either the N-tail or C-tail strongly inhibited the neurite-promoting activity of MK [26]. However their role appears to be to keep the two domains apart, since the C-terminal half (C-half) alone or even the C-domain shows a considerable degree of neurite-promoting activity [26, 27]. In the case of PTN, the C-tail itself is involved in its activity [28].

The C-half of MK has stronger heparin-binding activity than the N-terminal half (N-half) [27]. Indeed, there are two heparin-binding sites (Cluster-1 and -2) in the C-domain [25, 29]. Cluster-1 (K79, R81, and K102) is composed of basic amino acids in two *β*-sheets (Fig. **1**). Cluster -2 (K86, K87, and R89) is on a flexible loop [25]. Cluster 1 is present in PTN, but Cluster -2 is not. W69, conserved in the thrombospondin superfamily, is in a*β*-bulge and its chemical shift upon NMR spectroscopy is changed by the addition of a heparin oligosaccharide [25]. There are also other amino acids which are likely to be involved in heparin-binding as shown by chemical shift changes [25].

Because of important roles of carbohydrate recognition in MK activity to be mentioned later, the role of the two heparin-binding sites in MK activities has been systematically studied. Mutation of either of the two heparin-binding sites significantly reduces its neurite-promoting activity [26, 29]. However, binding to one of the MK receptors, receptor-like protein tyrosine phosphatase ζ(PTPζ) requires Cluster-1, but not Cluster-2 [30]. The enhancement of plasminogen activator activity in endothelial cells requires Cluster-2 rather than Cluster-1 [26]. 

MK forms a dimer through spontaneous association and also by cross-linking with transglutaminase, and heparin promotes the dimerization [31]. N-terminal half is required for the dimer formation [31]. Dimerization enhances MK activity to elevate the plasminogen activator activity of endothelial cells, and A41-P51 peptide, which contains the site required for dimerization by transglutaminase, inhibits the MK activity [31]. Furthermore, it has been suggested that upon dimerization Cluster-2 fuses to form a large binding site [25]. 

The C-tail is the site primarily recognized by rabbit anti-MK antibodies [27]. However, monoclonal antibodies directed at the C-domain or N-domain are available. 

### 2.3 Gene

**Fig. (3):**
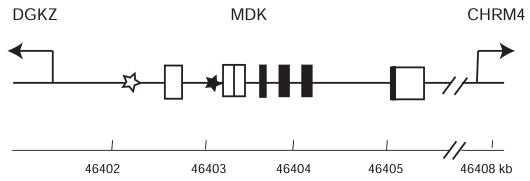
Organization of the human MK gene (*MDK*). Closed boxes show coding sequences in exons, and open boxes show noncoding sequences in exons. There are 3 non-coding exons. Usually, an MK mRNA has one of the non-coding exons. Open star, a retinoic acid-responsive element [41]; closed star, a functional WT1-binding site [43].

The human MK gene, *MDK*, is located on chromosome 11 at p11.2 [32], while the mouse MK gene, *Mdk,* is on chromosome 2 [33]. *MDK* is flanked by the diacylglycerol kinase z gene and muscarinic cholinergic receptor 4 gene [21, 34] (Fig. **3**). The human PTN gene, *PTN* is located on chromosome 7 at q33 [35], and is also flanked by a diacylglycerol kinase gene and a muscarinic cholinergic receptor gene [21, 34], indicating that *MDK* and *PTN* have evolved from a common ancestor through gene duplication [21].

*MDK* and *Mdk* span about 3 kb, while *PTN* is very large, about 130 kb [34, 36, 37]. *MDK* contains 4 coding exons and 3 non-coding exons [34] (Fig. **3**). In spite of the size difference, the intron / exon organization of *PTN* is similar to that of *MDK* [38]. There is a variant MK mRNA, which lacks an exon and encodes a truncated MK [39]. 

The expression of *MDK* and *Mdk* is controlled by a variety of factors. Consistent with the induction of its expression by retinoic acid, there is a functional retinoic responsive element in the promoter region [40, 41] (Fig. **3**). Glucocorticoid regulates the expression of MK through binding of its complex with the receptor to the promoter [42]. MK is strongly expressed in Wilms’ tumor cells [10], probably due to a loss of function of the tumor suppressor gene*WT1.* Indeed, a functional WT1-binding site is present in the promoter region of *MDK* [43] (Fig. **3**). MK expression after ischemic reperfusion injury and MK overexpression in malignant tumors can be explained at least partly by the presence of a hypoxia responsive element in the promoter [44]. MK expression induced by reactive oxygen species [45] indicates that there is still another region in the promoter responsible for the induction. 

### 2.4 *In Vitro* Activities and Signal Transduction

MK promotes various activities of target cells, such as growth [46-48], survival [49-52], migration [30, 53-55], neurite outgrowth [46, 47, 56] and protein production [57-60] (Table **1**). Among them, promotion of survival and that of migration are the central functions.

MK is a heparin-binding protein [61]. The recognition of acidic carbohydrate chains such as heparin is essential for MK activity, since heparin inhibits many function of MK [55, 56] and digestion with either heparitinase or chondroitinase abolishes responsiveness to MK [26, 56, 62]. Oligomers of two different structures bind to MK strongly. One is heparan sulfate trisulfated unit found in heparin, and the other is chondroitin sulfate E unit found in chondroitin sulfate E [63-66]. Affinities of the two structures to MK are at the similar level as shown by surface plasmon resonance, MK affinity chromatography and the inhibition of MK-dependent neurite outgrowth [63-66]. Related structures in chondroitin-4 sulfate, chondroitin-6 sulfate and chondroitin sulfate D showed little or no activity. 

PTPζ is the most established component of the MK receptor. It is involved in MK-dependent migration of neurons [30] and osteoblast-like cells [55] as well as promotion of cell survival by MK [67]. PTPζ is a proteoglycan and is a transmembrane protein with an extracellular chondroitin sulfate chain and cytoplasmic tyrosine phosphatase domain [68]. It binds to MK with the affinity of 0.58 nM, but after removal of chondroitin sulfate chain, the affinity decreases to 8.8 nM [30]. Therefore, MK is likely to bind mostly to the chondroitin sulfate portion of PTPζ. PTPζ is also a receptor of PTN [69].

The MK receptor is a molecular complex containing proteoglycans. Other proteoglycans with MK-binding activity are syndecans [70-72], glypican-2 [73], versican / PG-M [74] and neuroglycan C [75]. Neuroglycan C serves as an MK receptor for the promotion of neurites in oligodendrocyte precursor-like cells [75].

Low density lipoprotein receptor-related protein (LRP), integrin *α*_4_*β*_1_ and integrinα_6_β_1_ were identified as MK-binding proteins in embryonic mouse brain and shown to be components of the MK receptor complex [76, 77]. LRP binds to MK with an affinity of 3.5 nM. Related molecules LRP6 and apoE receptor 2 also bind to MK strongly [67].

Anaplastic lymphoma kinase (ALK) has been proposed as a receptor of MK [78, 79]. However, whether ALK binds to this molecule with high affinity is a matter of debate. Notch-2 is also an MK receptor upon epithelial-mesenchymal transition of immortalized keratinocytes [80].

LRP and integrin α_4_β_1_ or α_6_β_1_ have been found to bind strongly to each other [77]. PTPζ [77] and ALK (Muramatsu H *et al*, unpublished) also formed a complex with LRP and the integrins, while either of PTPζ and ALK could be in the complex (Muramatsu H *et al*, unpublished). Therefore it is likely that LRP and integrins form the core of the complex, and other molecules such as PTPζ and ALK are recruited to the complex. MK has been shown to increase complex formation of the receptor components [77]. There is evidence that neuroglycan C also functions in the receptor complex (Ichihara *et al*., unpublished).

The response to MK stimulation is increased tyrosine phosphorylation of cytoplasmic proteins such as paxillin [77], and a Src family kinase is involved in the process [55, 62]. A hypothesis explaining MK signaling upon the promotion of cell migration has been proposed [3, 77]. Among 2 reports on the action of PTPζ as the PTN receptor [81, 82], one supports the above hypothesis [81]. The downstream signaling system of the MK receptor includes MAP kinase and PI3 kinase [52, 55, 79]. Then, suppression of caspases [52, 83] or enhancement of Bcl-2 [84] occurs upon the promotion of cell survival. After its activation by MK, ALK phosphorylates insulin receptor substrate-1; the signal is transmitted by MAP kinase and PI3 kinase and results in the activation of NFκB [79]. Furthermore, the activities of STAT1α, STAT3 and STAT5 are regulated by MK [80, 85-87].

In addition to the signaling system mentioned above, MK is likely to function directly in the nucleus. Nucleolin, a shuttle protein moving between the cell surface and nucleus, has been identified as an MK-binding protein [88]. After binding to LRP, MK is internalized and transported to the nucleus by binding to nucleolin or laminin binding protein precursor [89, 90]. This nuclear transport of MK is important for MK-dependent cell survival [89]. Furthermore, MK in the nucleolus is involved in the synthesis of ribosomal RNA [91]. 

### 2.5 MK in Differentiation and Development 

MK is intensely expressed in the midgestation stage in mice [9, 70]. From its distribution, MK has been implicated in epithelial-mesenchymal interactions, neurogenesis and mesoderm remodeling [9, 70]. MK is generally more intensely expressed in the epithelial layer than mesenchymal layer [9]. Analysis of the development of the tooth germ *in vitro* has verified the role of MK in epithelial-mesenchymal interactions [92]. MK appears to inhibit excessive action of BMP, which induces translucent zone in the mesenchyme [92]. Furthermore, the mode of action of MK during epithelial mesenchymal interactions has been revealed by an artificial blood vessel model, in which collagen gel with smooth muscle cells is covered by blood vessel endothelial cells [60]. In this model, endothelial cells express MK, which acts on smooth muscle cells to induce IL-8 expression. IL8 then promotes the growth of endothelial cells. Thus MK plays a central role in the complex interactions of the epithelial and mesenchymal cells. During lung development, MK produced by the epithelial cells promotes the development of mesenchymal tissue [93]. Concerning the remodeling of the mesoderm, the introduction and expression of MK cDNA induces chondrogenesis by precursor cells [94]. Furthermore, MK induces adipocyte formation from 3T3-L1 cells [86]. On the other hand, MK decreases trabecular bone volume [95]. The role of MK in neurogenesis is dealt with in the subsequent section.

In spite of the various *in vitro* activities of MK, mice deficient in the MK gene show only few abnormalities in development [96]. The same is true for mice deficient in the PTN gene [97]. However, mice deficient in both the MK and PTN genes were born with a frequency 1/3 of that expected by Mendelian segregation [98], small in size [98] and frequently died before adulthood (Muramatsu H *et al*, unpublished). Furthermore, the female double knockout mice exhibited infertility [98]. These results have established that MK and PTN have important roles in reproduction and development.

MK and PTN are strongly expressed in granulosa cells in the ovary [98]. An analysis of the ovaries of the double knockout mice have revealed that follicular maturation is suppressed [98]. Deficit in follicular maturation caused by the lack of MK and PTN in granulosa cells has been concluded to be the primary reason of the female infertility.

In relation to the above-mentioned activity of MK, MK promotes the process of *in vitro *maturation of oocytes, fertilization and subsequent development to blastocysts [99, 100]. This activity of MK might be of practical importance. 

### 2.6 Neurogenesis 

In *Xenopus* embryos MK expression first occurs in neural anlagen, then increases when the neural tube is formed. Embryos injected with MK mRNA into the vegetal blastomeres develop abnormally and yield truncated tadpoles [101]. Histological examination have revealed that neurogenesis is enhanced and mesoderm differentiation is suppressed. The effect is stronger on injection into the dorsal than ventral blastomeres. Enhanced neurogenesis and suppressed mesoderm differentiation are also observed in excised animal caps treated with activin, when MK mRNA was preinjected. Based on the expression of neural markers, the induced neural tissue has been concluded to be anterior [101]. 

The zebrafish has two MK genes due to gene duplication: Mdka is expressed in the paraxial mesoderm [102], and Mdkb in the neural plate [103]. Mdka is involved in determining whether a population of midline cells become floor plate cells, which make up the basal portion of the neural tube and develop into the corresponding portion of the spinal cord [102]. Mdka is a factor, which determines the cell fate of the precursor cells either to floor plate cells or to notochord cells. Mdkb is required for the specification of cell fates at the neural plate border and for the development of neural crest cells and sensory neurons [103].

In relation to the above finding, antisense oligonucleotide to MK inhibits the conversion of mesenchyme to neuroectoderm in cultured tail bud cell of chiken embryos [104]. Furthermore, mice deficient in the MK gene show moderate auditory deficits, and mice doubly deficient in both MK and PTN show severer deficits [105, 106].

At the midgestation stage in mice and rats, MK is strongly expressed in the basal layer of the cerebral cortex, which is rich in neural precursor cells including neural stem cells, and also in radial glial processes, which are extended processes derived from neural stem cells [9, 107, 108]. Differentiated neurons migrate along radial glial processes. MK is also strongly expressed in neural precursor cells in culture and is involved in their growth and survival [109]. Thus, neural precursor cells from MK-deficient mice grew poorly on dishes coated with poly-L-lysine but grew well on dishes coated with MK [109].

MK is strongly expressed in the rat cerebellum 7 days after birth [110]. MK is probably involved in migration and neurite outgrowth in these cells.

### 2.7 Repair and Inflammation

MK is generally expressed in response to tissue damage such as ischemic injury in the brain [11], heart [111], kidney [59] and blood vessels [54]. MK can promote the repair of damaged tissue by enhancing the survival of injured cells [111-114]. Anti-bacterial activity of MK [115] would also be beneficial to tissue repair. 

On the other hand, MK promotes inflammatory responses by enhancing the migration of inflammatory leukocytes such as neutrophils [53] and macrophages [54], increasing synthesis of chemokines [59], and suppressing the production of regulatory T cells [87]. An excessive inflammatory response is harmful to tissue repair.

The phenotype of MK-deficient mice is helpful in determining whether MK induced upon injury is beneficial or harmful to tissue repair (Table **2**). For example, MK is concluded to promote the repair of the heart tissue after ischemic injury, since infarct size is greater in MK-deficient mice than wild-type mice [111]. Furthermore, MK-deficient mice die more frequently from ischemic heart damage [111]. However, MK is concluded to be harmful to the kidney after ischemic renal injury, since the damage to the renal tissue is more extensive in wild-type mice than MK-deficient mice [59]. Similar phenomena have been observed in other inflammatory diseases (Table **2**).

### 2.8 Cancer

MK is overexpressed in most of human malignant tumors, such as carcinomas of the oral cavity [116], esophagus [117], stomach [117], colon [117], liver [117], pancreas [118], lung [119], urinary bladder [120], prostate [121], breast [122], uterus [123, 124] and ovary [125], osteosarcoma [126], soft tissue sarcoma [127], neuroblastoma [128], astrocytoma [129 ], meningioma [130], and acute lymphoblastic leukemia [131]. The overexpression is usually found in about 80 % cases of respective tumors, as in hepatocellular carcinoma [117], colon carcinoma [117], lung carcinoma [119] and prostate carcinoma [121]. MK overexpression is often detected even in the precancerous stage [132, 133]. In the carcinogenesis of colon cancer, MK becomes detectable in the moderate and severe stages of dysplasia [132]. A gradual increase in MK expression accompanying the progression of disease has also been observed [121, 129]. Furthermore, the strong expression of MK in tumors is often correlated with a poor prognosis [116, 118, 120, 121, 124, 128, 129]. One reason for the overexpression of MK in tumors is the presence of a hypoxia responsive element in the MK promoter [44]. Loss of activity of the product of a tumor suppressor gene also leads to MK overexpression [43, 134].

Overexpression of MK has been concluded to contribute to the malignant properties of cancer cells. As an example, transfection with MK cDNA transforms NIH3T3 cells [135]. Furthermore, MK overexpression is correlated with resistance to chemotherapy [136, 137]. Generally speaking, MK is considered to be involved in tumor invasion by promoting the growth, survival [138], migration and angiogenic activity [139] of tumor cells. 

Because of its general overexpression in human cancers, MK has been the subject of intense research as a tumor marker. Indeed, serum or plasma levels of MK increase in the majority of patients with various malignant tumors [140-149]. The urinary MK level is also useful for detecting cancer in certain cases [150]. Serum MK levels are especially helpful in the detection of α-fetoprotein-negative hepatocellular carcinoma [146]. Furthermore, high levels of MK in serum or plasma have been correlated with poor prognosis [145, 147, 148]. Since serum MK levels also increase in patients with rheumatoid arthritis [151], Alzheimer’s disease [152] and chronic heart failure with a high risk of cardiac events [153], the utility of MK as a tumor marker may be confined to apparently healthy subjects.

A truncated MK produced by alternative splicing may be of interest as a tumor marker because of its tumor-specific expression, occurrence in lymph node metastasis and contribution to the malignant characteristics [39, 154-157]

MK is also promising as a molecular target to treat malignant tumors, and the MK promoter can be used for the tumor-specific expression of a gene for curative purposes. These points will be mentioned in a later section in relation to the treatment of glioblastomas.

### 2.9 Other Diseases

MK is correlated with hypertension, since it is a regulator of the renin-angiotensin system [45, 158]. Upon 5/6 nephrectomy, MK is expressed in the lung, and induces the expression of angiotensin-converting enzyme [45]. Hypertension is induced after the nephrectomy in wild-type mice but not significantly in MK-deficient mice [45].

MK is also involved in the secretion of insulin. A complex of apolipoprotein A-V and MK is internalized and induces insulin secretion in rat pancreatic *β*-cells [159]. A possible association of MK with diabetes is an interesting subject to be examined. 

## 3 TREATMENT OF DISEASES OF THE CENTRAL NERVOUS SYSTEM

### 3.1 Neuronal Death Upon Cerebral Infarction

Delayed neuronal death occurs around an area of the infarct referred to as the ischemic zone and upon the sudden opening of blood stream after an infarction, and is a serious complication of cerebral infarction.

MK expression upon cerebral infarction was first observed in the rat [11]. Experimental infarction was produced in the left cerebral cortices of rats by permanent occlusion of the middle cerebral artery. Immunohistochemical staining has revealed MK expression in edematous and periischemic areas around the infarct. The infarct, characterized by the shrinking or necrosis of neurons, does not express MK. The expression is found as early as Day 1 after occlusion, is intense till Day 4, becomes weak on Day 7, and disappears on Day 14. The cells expressing MK have been identified as astrocytes [160]

MK is also expressed after transient forebrain ischemia in rats [161]. Before the ischemic insult, a basal level of MK is observed in hippocampal neurons notably CA1 pyramidal neurons, cortical neurons and the choroid plexus. Following 20 min of forebrain ischemia and reperfusion, MK expression decreases till Day 2. A decrease is observed in both the number of MK-expressing cells and the intensity of expression in individual cells. On Day 4 after the insult, many scattered cells around the CA1 pyramidal layer intensely express MK. These cells have been identified as reactive astrocytes.

The induction of MK expression after ischemia implied a role for MK in the prevention of neuronal death. Indeed, the intraventricular injection of MK was found to ameliorate delayed neuronal death in the hippocampus after transient forebrain ischemia in gerbils [113]. The ischemia was achieved by occluding the bilateral common carotid arteries for 5 min. Examination after 7 days revealed extensive neuronal death in the CA1 region of the hippocampus. The number of surviving cells in the CA1 region was 17±25 / mm after ischemia reperfusion, and 265±38 / mm without the treatment (sham operation). The injection of MK solution immediately before the occlusion greatly improved the survival of hippocampal neurons. After the administration of 2 *μ*g MK, the number of surviving cells on Day 7 after ischemia increased to 254±68 / mm. Consistent with this result, the number of TUNEL-positive cells in the CA1 region after transient ischemia decreased upon the injection of MK. However, there were two problems. First, MK administered 2 h after ischemia reperfusion was not significantly effective. Second, the neurons survived to Day 7 and then progressively died, and the effect of MK became insignificant on Day 28.

Cell death in the periischemic area after infarction is suppressed by postischemic gene transfer and expression of MK [162]. Cerebral infarction was produced by photochemical occlusion of the distal middle cerebral artery in spontaneously hypertensive rats. Ninety minutes after the occlusion, an adenoviral vector with MKcDNA (adeno-MK) was injected into the lateral ventricle on the ischemic side. Two days after the infarction, the infarct was significantly smaller in rats transfected with adeno-MK than in those transfected with a control adenovirus: the reduction in infarct volume on the transfer of adeno- MK was 41 %. At the same time point, TUNEL-positive cells in the periischemic area were reduced to about 20 % of the control value by treatment with adeno-MK. 

The reduction in infarct volume by adeno-MK was found even 7 days after infarction [163]. Treatment with adeno-MK does not significantly alter the number of infiltrating macrophages and proliferating neural precursor cells. However, the number of neural precursor cells migrating toward the infarct is increased by the treatment. All these results clearly indicate that MK expression induced by ischemic injury in astrocytes suppresses neuronal death. Although MK helped to prevent neuronal death after postischemic treatment in one of the two studies mentioned above, the difference is probably due to the difference in the animal model used and not to the method of supply or expression of MK. MK is expected to be clinically helpful to reduce infarct volume by rescuing neurons in periischemic regions. As a supportive evidence, MK administered shortly after ischemia reperfusion injury suppresses the death of heart cells in the mice [111].

A neuroprotective effect of MK has also been clearly demonstrated by another study. Temporal lobe epilepsy is a major type of epilepsy, and characterized by neuronal loss in subregions of the hippocampus. An excellent animal model of temporal lobe epilepsy is the seizure caused by an intracerebroventricular injection of kainic acid [164]

Twenty-four hours after kainic acid is injected, basal levels of MK immunoactivity present in hippocampal pyramidal neurons decreases, while the number of MK-positive cells in the stratum lacunosum moleculare of CA3 markedly increases. The MK-positive cells are astrocytes and not microglia [164]. 

Administration of MK together with kainic acid is able to suppress the seizures [164]. The seizures lasted for mean 1,201 sec without treatment, and for mean 319 sec in mice treated with 0.4 *μ*g of MK. When the intensity of the seizures was scored, the maximum score was 5.0 when jumping, circling, or rolling was observed. The mean score was 4.2 without treatment, and was 1.7 with 0.4 *μ*g of MK. 

The extensive degeneration of neurons observed in hippocampal subregions after the kainic acid treatment is significantly reduced by the administration of MK. The effect was strongest in the hilus, in which the number of neurons was reduced to about 15 % of that before the treatment with kainic acid. In the mice administered with MK, however, about 82 % of the neurons in that region survived. A majority of neurons also survived in CA3 after the administration of MK. Loss of GABAergic interneurons after kainic acid treatment was also significantly suppressed in the strata pyramidale (68 % improvements) and radiatum (45 %) of CA3 and molecular layer (27 %), granular cell layer (29 %) and dentate hilus (33 %) of the dentate gyrus [164].

These results illustrate the strong neuroprotective effect of MK. The effect was found at low doses: the maximum dose of MK was 0.4 *μ*g per mouse. The effect of MK was dose-dependent, and a higher dose of MK might achieve further protection.

### 3.2 Multiple Sclerosis

Multiple sclerosis is an autoimmune disease characterized by inflammatory demyelination in the central nervous system. Experimental autoimmune encephalitis (EAE) is an animal model of multiple sclerosis. Recent studies have revealed that MK is involved in the etiology of EAE [87]. 

EAE induced by the injection of myelin oligodendrocyte glycoprotein peptide is less severe in mice deficient in the MK gene than in wild-type mice [87]. Supplying MK to the deficient mice abolishes the suppression of EAE. Inflammatory cells infiltrate the spinal cord to much lesser extent in the deficient mice, but on the supply of MK the infiltration resumes [87].

The number of CD4+ CD25+ regulatory T cells in lymphoid tissues at the onset of the disease is greater in MK-deficient EAE mice than wild-type EAE mice [87]. The most plausible explanation of this finding is that MK suppresses the expansion of regulatory T cells. This point has been verified by using cultured spleen cells: MK at the concentration of 20 – 100 ng / ml effectively suppresses the expansion. Furthermore, in MK-deficient mice, Th1 and Th17 cells, which mediate autoimmune disease, are suppressed as shown by the levels of cytokines produced by these cells.

These results indicate that MK suppresses regulatory T cells, thereby increasing Th1 and Th17 activities, and thus plays a key role in the onset of EAE. Therefore, suppression of MK activity is expected to suppress EAE. Indeed, an aptamer directed at MK has been found to exhibit such activity [87].

RNA aptamers have high specificity and affinity for target molecules and are reported to have advantages over antibodies. The RNA aptamer used for the study was a 49-mer, and was stabilized with ribose-2’ modifications as well as cholesterol and inverted dT tags at the 5’ and 3’ ends. The Kd of the aptamer to MK was estimated to be 0.9 nM. The anti-MK aptamer induced expansion of regulatory T cells* in vitro*. This aptamer was i. p. injected to mice every other day from the start of immunization with myelin oligodendrocyte glycoprotein peptide. The treated mice showed a retarded onset of EAE and lower clinical scores. The highest dose of the aptemer, 15 mg /kg, had the greatest effect. The aptamer was effective even when it was administered after the onset of EAE [87].

These results indicate that anti-MK therapy is a promising treatment for multiple sclerosis. RNA aptamers might be the best choice for treating pathological regions in the spine. Low molecular weight inhibitors of MK, once they are developed, should also be effective. 

### 3.3 Glioblastoma

Glioblastoma or glioblastoma multiforme is a common form of brain tumor known for an aggressive nature and very poor prognosis. Glioblastoma belongs to tumors derived from astrocytes, collectively called astrocytomas. MK expression has been found to increase during the progression of human astrocytomas [129]. Thus, MK mRNA or protein is scarcely expressed in control brain tissue, weakly in low grade glioma, moderately to strongly in anaplastic astrocytoma and strongly in glioblastoma. MK overexpression was found in all of 9 glioblastoma specimens. The overexpression of MK appears to contribute to the malignant phenotypes of glioblastoma such as enhanced angiogenesis. 

The strong expression of MK in glioblastoma is of significance for treatment. Indeed, an MK-promoter-based conditionally replicating adenovirus (Ad-MK) exhibits strong oncolytic effects on malignant glioma cells, but not normal brain cells [165]. Ad-MK was constructed by placing a 0.6-kbp fragment from the MK promoter in the 5’ region of the E1A gene in the adenovirus genome [166]. The 0.6-kbp fragment exhibits 2 orders of higher promoter activity in malignant glioma cells than in normal brain cells. Furthermore, the injection of Ad-MK completely eradicates xenografts of malignant glioma [165]. 

Ad-MK also suppresses the growth of xenografts of pancreatic carcinoma [167] and bladder carcinoma [168]. Ad-MK constructed in a different manner is effective for purging bone marrow tumors [169]. The MK promoter is suitable for tumor-specific gene expression because of its strong activity, ability to function in a broad range of tumor cells and lack of activity in most non-cancerous cells. For example, the promoter fragment acts as efficiently as the α-fetoprotein promoter in hepatocarcinoma cells [170].****

The tumor-specific nature of the MK promoter was clearly illustrated in the following experiment. The thymidine kinase gene was placed under the control of the MK promoter or cytomegalovirus (CMV) promoter in a replication-deficient adenoviral vector [171]. On the local administration of either adenoviral construct followed by gancicrovir treatment, the growth of xenografted Wilms’ tumor was suppressed almost completely. However after systemic delivery and ganciclovir treatment, the construct with the CMV promoter killed mice, whereas the construct with the MK promoter did not [171]. This is because thymidine kinase under the control of the CMV promoter was expressed in the liver and exhibited hepatic toxicity in the presence of ganciclovir. The MK promoter is inactive in the liver, and no sign of hepatic toxicity was found after transfection of the construct with the MK promoter. 

All these results illustrate the utility of the MK promoter and its fragment for the tumor-specific expression of oncolytic viruses or therapeutic proteins. Further refinements of the methods may lead to novel therapeutic treatments for glioblastoma. 

Strategies to suppress MK expression were not employed to treat glioblastoma in experimental models. However, siRNA or antisense oligoDNA to MK has been used to suppress the growth of other tumors. Thus, injection of antisense oligoDNA to mouse MK into mouse colorectal carcinoma pregrown in nude mice with the aid of atelocollagen effectively inhibits tumor growth [48]. siRNA to human MK suppresses the growth of human prostate carcinoma implanted in nude mice only moderately. However, a combination of the siRNA and taxol abolishes the tumor growth almost completely, at a taxol concentration at which only a partial inhibition of tumor growth is attained [172]. Therefore, even at the present stage of development, siRNA to human MK augments the effect of chemotherapy. The relatively less effect of siRNA to human MK to inhibit tumor growth might be partly due to the presence of host MK, which is of mouse origin, and might not be inhibited by the reagent. Indeed the lung metastasis of MK-negative Lewis carcinoma inoculated into the leg is much less extensive in MK-deficient mice compared to that in wild-type mice [173]. Nevertheless, an antisense oligoDNA to human MK effectively suppresses the growth of xenografted human hepatocarcinoma, when delivered by nanoliposomes [174]. 

Thus, methods of inhibiting the expression of MK should be considered as an option for treating glioblastoma. Suppression of PTN and ALK expression has already been employed as means to treat glioblastoma, and promising results have been obtained in animal experiments [175].

In addition to siRNA or antisense oligoDNA, drugs which inhibit the action of MK should be tested against glioblastoma, when they become available.

### 3.4 Neurodegenerative Diseases and Neuropsychiatric Diseases

MK has been found to accumulate in senile plaques in the brain of patients with Alzheimer’s disease. Senile plaques and neurofibrillary tangles are the two major pathological hallmarks of Alzheimers’s disease and closely associated with the pathology of the disease. 

Immunohistochemical staining revealed the presence of MK in the senile plaques in cerebral cortex of all 8 patients with Alzheimer’s disease but not in the control specimens from 3 individuals without neurological diseases [176]. In consolidated plaques, the core was more intensely stained than the periphery. After formic acid treatment, the intensity of the staining was increased, and almost all amyloid *β*-peptide plaques were stained by the anti-MK antibody. Western blotting confirmed that the MK-like immunoreactivity detected immunohistochemically was MK itself [176]. Elevated serum levels of MK are also detected in about half of patients with Alzheimer’s disease [152].

MK has been found to inhibit cytotoxicity [177] and polymerization [178] of amyloid *β*-peptide. Therefore, MK appears to be produced to counteract the deposition of amyloid *β*-peptide plaques. Indeed, the deposition of amyloid *β*-peptide plaques derived from transgene was more extensive in mice deficient in the MK gene than in wild-type mice [179]. Furthermore, MK promoted the migration of microglias, which are involved in the clearance of amyloid *β*-peptide plaques [179]. Thus, MK is likely to counteract the deposition of amyloid *β*-peptide plaques by both directly binding to amyloid *β*-peptide and promoting the migration of microglias.

MK immunoreactivity is also present in neurofibrillary tangles in brains of patients with parkinsonism-dementia complex of Guam [180]. MK is deposited in glial cytoplasmic inclusions in patients with multiple system atropy [181]. MK expression is also intense in the prefrontal cortex of chronic alcoholics [182]. In the rat hippocampus, chronic administration of morphine and yohimbine also increases MK expression [183]. In all these cases MK can be considered to be induced to counteract neural damage. 

In MK-deficient mice, abnormal brain function was found only before adulthood at the initial phase of analysis [96]. Recent studies however, have revealed that dopamine D1 and D2 receptors and dopamine itself are decreased in the striatum of adult MK-deficient mice [184]. MK promotes the survival of mesencephalic neurons in culture [50]. Thus, MK deficiency may have resulted in the dysfunction or decrease of dopaminergic neurons in these mice. NMDA receptors and NMDA-receptor-related amino acids in the hippocampus and frontal cortex are not altered in the deficient mice. 

Prepulse inhibition has been found to decrease in the hypodopaminergic MK-deficient mice [184]. Decreased prepulse inhibition is observed in patients with neuropsychiatric disorders including schizophrenia and autistic disorders. Treatment with antipsychotic, namely haloperidol or clozapine, restores prepulse inhibition to a level indistinguishable from that of wild-type mice [184]. Furthermore, MK-deficient mice exhibit reduced numbers and longer periods of social contact with other mice. Thus MK-deficient mice may be considered as a model of a subtype of schizophrenia characterized by a hypodopaminergic state. 

The design of new treatments for the diseases mentioned in this section requires more basic research. The administration of MK to treat neurodegenerative diseases such as Alzheimer’s disease, which has a long clinical process, might not be practical. A more likely application is the enhancement of the growth and survival of a certain cell population upon regenerative therapy. MK has recently been shown to promote the growth of embryonic stem cells [185]. Furthermore, MK expressed in neural precursor cells promotes their growth and survival [109]. In addition, full understanding of the MK promoter may aid the development of new drugs for treating neurodegenerative diseases, since such knowledge may make it possible to screen compounds which activate the MK gene in brain cells but not in precancerous cells. Concerning improvement of the hypodopaminergic state, it will be important to know whether the supply of MK in the adult is effective for the purpose.

## 4 DRUG DEVELOPMENT

### 4.1 MK Protein

MK is useful to suppress neuronal death especially upon ischemic injury [113]. MK has been produced as a recombinant protein in yeast [186], baculovirus [56], L cells [46] and *Escherichia coli* [88], and also chemically synthesized [187]. So far the most widely used preparation to promote cell survival is the one produced by yeast. Yeast-made MK is effective to promote survival of neurons [113] and heart cells [111, 112]. It can be obtained in relatively large amounts and at least the major portion is likely to be properly folded. However, aberrant glycosylation, especially mannosylation, might occur under certain conditions. Production in mammalian cells is an alternative choice. However, the cells should be rigorously checked as to whether they produce MK of their own even in small amounts. Contamination with MK from different species may not be easily removed during purification. MK produced by L cells is effective to promote the survival of photoreceptor cells exposed to constant light [114].

MK has 5 disulfide linkages [24]. Therefore, attention should be paid so that only the molecule with correct disulfide linkages is used. In the chemical synthesis of MK, half molecules (N-half and C-half) were separately synthesized. These half molecules were fractionated by HPLC to isomers with different disulfide bridges. After structural analysis, only the ones with correct disulfide bridges were connected by chemical means to yield the MK molecule [187]. When the scrambling of disulfide linkages occurs extensively in a preparation of recombinant MK, a similar approach can be used to produce the recombinant protein, since chymotrypsin cleaves MK into halves [188]. 

Since MK is an adhesive protein, care is needed not to lose it through absorption to vessels, especially upon storage. Our recommended conditions for storage are -80 ^0^C in 50 mM phosphate buffer pH 6.8 containing 1 – 0.5 M NaCl with an MK concentration of more than 0.1 mg/ml [66]. Repeated freezing and thawing should be avoided. With higher MK concentrations, storage under lower NaCl concentrations may be possible. High concentrations of NaCl can be removed by dialysis prior to usage. To dilute the stock solution of MK, vessels are preferably coated with silicon.

Potential side effects of MK can be enhanced inflammation and promotion of carcinogenesis. It is proper to mention that response to MK is different in individual organs, and enhanced inflammation was not reported upon treatment of ischemic injury in the brain and heart by MK [111, 113]. Concerning promotion of carcinogenesis, administration of MK for short time period is expected to be harmless, since MK is an endogenous factor induced after injury. More caution is required for prolonged application of MK. 

### 4.2 MK Inhibitors

Various MK inhibitors are now used to treat malignant and inflammatory diseases in experimental models. siRNA and antisense oligoDNA are much frequently used [48, 172, 174, 189-192] (Table **3**), and might be applied to the treatment of glioblastoma. 

For the development and evaluation of other inhibitors, a rapid assay system is essential. An assay based on inhibition of the MK-dependent migration of UMR106 osteoblast-like cells was recently developed [193]. In this assay, migrating cells are colorimetrically determined. Although many cells, which respond to MK strongly, are primary cultured cells, UMR106 cells are an established cell line, and time-consuming procedures of cell preparation can be avoided. In the assay, MK is provided as a substratum-bound form. While soluble MK elicits various cellular responses, its level of activity is usually 2-3 times of the preexisting level. Substratum-bound MK often evokes a stronger response. Thus, the inhibition of substratum-bound MK is generally more convenient for testing MK inhibitors. Although aptamers to MK were screened by this assay, the selected aptamer inhibited activity of soluble MK to suppress the expansion of regulatory T cells [87]. Other candidate assays to screen MK inhibitors may be the inhibition of MK-dependent survival of embryonic neurons from MK-deficient mice and inhibition of MK-induced cytokine production by human leukocytes.

**Fig. (4):**
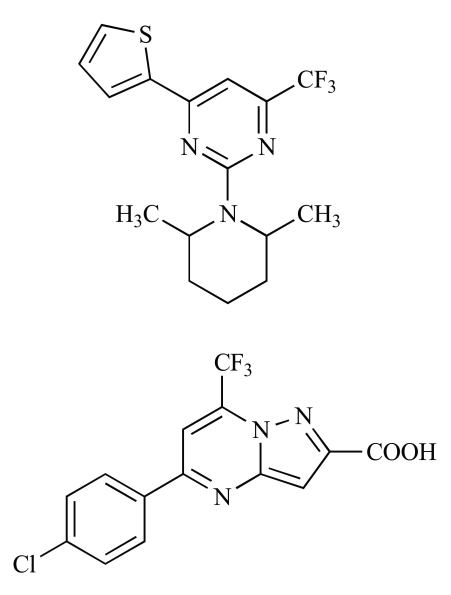
Structures of two compounds initially selected as low molecularweight MK inhibitors [193]. Upper one, PubChem 4603792; lower one, a related compound.

An aptamer to MK has already been used to treat EAE in mice, and might become clinically useful [87]. On the other hand, low molecular weight compounds with MK inhibitory activity are at the initial stages of development. Because of the presence of libraries of drug-like compounds such as the Zinc library and efficient *in silico* docking programs, it is not difficult to search for candidate MK inhibitors in drug-like compounds. Selected candidate compounds can then be tested for actual MK inhibitory activity. Indeed, in such a study [193], two compounds with MK-inhibitory activity were found; they did not exhibit adverse cytotoxicity. One compound was 2-(2,6-dimethylpiperidin-1-yl)-4-thiophen-2-yl-6-(trifluoromethyl)pyrimidine (PubChem 4603792) and the other had a similar structure (Fig. **4**). Interestingly, both had a trifluoro residue. Although their MK inhibitory activities were not strong, such compounds could be used as a mother compound to develop clinically effective MK inhibitors. Conversion of a methyl group to a carboxyl group was even proposed as a means to produce a more potent inhibitor [193].

In the study mentioned above [193], only a fraction of the Zinc library was *in silico* surveyed, and among numerous compounds with potential MK inhibitory activity, only those available from a company, Chem. Div., were used to test actual MK inhibitory activity. Thus a more systematic study might yield much better inhibitors. Furthermore, only compounds with expected strong affinity for a heparin-binding site in the C-half were searched in the above study. The requirement of a key arginine residue in the heparin-binding site for MK activity was indeed verified by site-directed mutagenesis [29]. However, other parts of the molecule are also required for MK activity. As an example, N-half of MK is necessary for MK dimerization [31], which is essential for part of the MK activity. Indeed, N-terminal portion of the molecule was used as a dominant-negative MK inhibitor, to analyze its role in neurogenesis in the zebrafish [194]. In this connection, a segment of MK in the N-domain is conserved between human MK, *Xenopus* MK and zebrafish Mdka (Fig. **1**), and might play a critical role in some MK activities. Furthermore, some monoclonal antibodies reactive with the N-domain inhibited MK activity (Sakuma *et al*., unpublished). Therefore, low molecular weight compounds which bind to the N-domain should be also screened.

Fragments from MK receptors also have MK inhibitory activity. Notably, a peptide derived from β_1_-integrin moderately inhibited MK activity [193]. Furthermore the N-terminal half of the second ligand-binding domain of LRP binds to MK strongly [195]. Expression of this protein fragment in G401 Wilms’ tumor cells suppresses their growth and colony formation in soft agar [195]. 

Although polyclonal antibodies have been reported to inhibit the growth of tumor cells such as G401 Wilms’ tumor cells [47] and osteosarcoma cells [126], no monoclonal antibody was able to do so, to the best of my knowledge. An aptamer with the ability to inhibit EAE did not strongly suppress the growth of tumor cells either (unpublished results). MK in tumor cells might form a stable complex with cell surface receptors or also act intracellularly, and only a fraction of antibodies are able to inhibit MK under such conditions. Further efforts to obtain a monoclonal antibody to inhibit MK in tumor cells might yield promising results.

Both chondroitin sulfate E and heparin are known to inhibit various MK activities. Heparin strongly binds to many kinds of proteins, while the reactivity of chondroitin sulfate E is more limited. Therefore, chondroitin sulfate E might be more suitable as a mother material to design a carbohydrate-based MK inhibitor. Chondroitin sulfate E itself was shown to inhibit antibody-induced arthritis [192]. It is necessary to determine the minimum length of chondroitin sulfate E repeating units to attain strong inhibition. In the case of heparin, a fragment of apparent molecular weight more than 7,000 is necessary to exhibit strong inhibitory activity [63]. A similar result is expected for chondroitin sulfate E. However, some portion of the carbohydrate might be required only to maintain the three dimensional structure of the chain. If so, chemical modification may replace some of the oligosaccharide chain. Furthermore, it should be examined whether the introduction of another sulfate group to a specific position increases the binding to MK without affecting the specificity. In this context, sulfation at 3-position of glucuronic acid does not appear to increase the binding to MK. This is because natural chondroitin sulfate E, which has considerable amounts of glucuronic acid 3-sulfate, binds to MK agarose with an affinity similar to enzymatically synthesized chondroitin sulfate E, which should lack the additional sulfate group [65]. 

Finally, side effects of MK inhibitors might be restricted, because of limited expression of MK in adult tissue. Still, based on current knowledge on MK, we should remember the possibility that MK inhibitors may enhance susceptibility to infection and tissue degeneration and hinder tissue regeneration.

## 5 CONCLUSION

Both MK and MK inhibitors are expected to be useful for treatment of diseases in the central nervous system. Many works remain to be done about development of clinically applicable MK inhibitors.

## Figures and Tables

**Table 1:** Activities of MK to Target Cells

Target cells	Activities
Fibroblasts	Growth [46], Contraction of collagen gells [196],
	Synthesis of collagen and glycosaminoglycans [58]
Keratinocytes	Growth [197]
Endothelial cells	Fibrinolytic activity [57]
Renal epithelial cells	MIP-2 production [59]
Smooth muscle cells	Migration [54], IL8 production [60]
Myoblasts	Clustering of acetylcholine receptor [198]
Osteoblast-like cells	Migration [55]
Embryonic neurons	Survival [49-52], Neurite outgrowth [46, 47, 56]
	Migration [30]
Neural precursor cells	Growth [109], Survival [109]
Oligodendrocyte	Neurite outgrowth [75]
	precursor-like cells
Neutrophils	Migration [53]
Macrophages	Migration [54]
Tumor cells	Growth [47, 48, 126]

**Table 2:** Phenotypes of MK-Deficient Mice in Relation to Diseases

Diseases	Phenotypes
Heart failure	Enhanced damage and decreased survival upon ischemic heart damage [111]
Peripheral nerve injury	Delay in both degeneration and regeneration [199]
Hepatitis	Decrease in liver regeneration upon partial hepatectomy [200]
Nephritis	Decreased renal damage upon ischemia [59], chemotherapy [201] and diabetics [202]
Hypertension	Decreased hypertension after 5/6 nephrectomy [45]
Rheumatoid arthritis	Decreased arthritis upon antibody-induced arthritis [151]
Multiple sclerosis	Decreased neurological symptom upon EAE [87]
Restenosis	Decreased neointima formation after ischemic injury [54]
Adhesion after surgery	Decreased adhesion [203]
Cancer	Decreased lung metastasis [173]
Osteoporosis	Increased trabecular bone formation [95]
Alzheimer’s disease	Increased deposition of amyloid *β*-peptide plaques [179]

**Table 3:** Inhibitors of MK in Treatment of Diseases Upon Experimental Systems

Inhibitors	Effective diseases
Antisense oligoDNA	Cancer [48, 174], Nephritis [189],
	Neointima formation [191]
siRNA	Cancer [172], Neointima formation [190],
	Adhesion after surgery [192]
Aptamer	EAE [87]
Chondroitin sulfate E	Antibody-induced arthritis [192]

## ABBREVIATIONS

ALK: = Anaplastic lymphoma kinase
CMV: = Cytomegalovirus
EAE: = Experimental autoimmune encephalitis
HB-GAM: = Heparin-binding growth-associated molecule
LRP: = Low density lipoprotein receptor-related protein
MK: = Midkine
PTN: = Pleiotrophin
PTPζ: = Receptor-like protein tyrosine phosphatase ζ
